# Prevention of stent migration of covered self-expandable metal stents in distal malignant biliary obstruction: a review of literature

**DOI:** 10.1093/gastro/goaf058

**Published:** 2025-06-29

**Authors:** Jung Won Chun, Woo Hyun Paik, Sang Myung Woo, Jin Ho Choi, In Rae Cho, Woo Jin Lee, Ji Kon Ryu, Yong-Tae Kim, Sang Hyub Lee

**Affiliations:** Center for Liver and Pancreatobiliary Cancer and Research Institute, National Cancer Center, Goyang-si, South Korea; Department of Internal Medicine and Liver Research Institute, Seoul National University Hospital, Seoul National University College of Medicine, Seoul, Korea; Center for Liver and Pancreatobiliary Cancer and Research Institute, National Cancer Center, Goyang-si, South Korea; Department of Internal Medicine and Liver Research Institute, Seoul National University Hospital, Seoul National University College of Medicine, Seoul, Korea; Department of Internal Medicine and Liver Research Institute, Seoul National University Hospital, Seoul National University College of Medicine, Seoul, Korea; Center for Liver and Pancreatobiliary Cancer and Research Institute, National Cancer Center, Goyang-si, South Korea; Department of Internal Medicine and Liver Research Institute, Seoul National University Hospital, Seoul National University College of Medicine, Seoul, Korea; Department of Internal Medicine, National Medical Center, Seoul, Korea; Department of Internal Medicine and Liver Research Institute, Seoul National University Hospital, Seoul National University College of Medicine, Seoul, Korea

**Keywords:** malignant biliary obstruction, covered SEMS, biliary drainage, anti-migration, endoscopic retrograde cholangiopancreatography

## Abstract

Distal malignant biliary obstruction (dMBO) is a common complication of advanced malignancies, particularly pancreatic cancer and biliary tract cancer, requiring biliary drainage to relieve symptoms. Endoscopic drainage using self-expandable metal stents (SEMS) is widely preferred due to improved long-term patency compared with plastic stents. However, the choice between fully covered SEMS (FCSEMS) and uncovered SEMS (UCSEMS) remains controversial, primarily due to migration risks associated with FCSEMS. Recent advances in stent design, such as anchoring flaps, flared ends, and anti-migration coatings, have been developed to improve FCSEMS stability. Additionally, techniques incorporating double-pigtail plastic stents as internal or external anchors have demonstrated significant reductions in migration rates. This review examines the current literature and evaluates various anti-migration strategies for FCSEMS, highlighting the clinical efficacy and challenges associated with each approach. Understanding these innovations is crucial for optimizing stent selection and improving patient outcomes in dMBO.

## Introduction

Distal malignant biliary obstruction (dMBO) commonly occurs in patients with malignancies, such as pancreatic cancer, bile duct cancer, ampullary tumors, and metastatic disease, leading to obstructive jaundice, cholangitis, and pruritus [1]. These symptoms adversely affect patients’ quality of life, interfere with chemotherapy in unresectable cancers, and reduce survival rates. Effective biliary drainage is essential for the management of unresectable malignant biliary obstruction (MBO) and endoscopic retrograde cholangiopancreatography (ERCP) is considered the standard of care [2].

The optimal type of stent for maintaining biliary patency remains a point of debate due to complications, such as stent occlusion and migration. Self-expandable metal stents (SEMS) have been widely adopted due to their superior long-term patency compared with plastic stents (PS) [3, 4]. SEMS can be broadly divided into three types: uncovered SEMS (UCSEMS), fully covered SEMS (FCSEMS), and partially covered SEMS (PCSEMS). The covering membrane can prevent tumor ingrowth or epithelial hyperplasia, thereby extending the patency duration of the stent [5]. Despite this, previous reports have shown no significant prolongation of stent patency by depending on the covering membrane [6–8]. This may be due to their higher rates of stent migration compared with UCSEMS.

Several studies have reported various approaches to prevent stent migration, including partial covering, flared ends, anchoring flaps, banding structures, and higher radial force, although it remains unclear which design is superior [9–14]. However, these stents sometimes cause difficulty in stent removal due to tissue ingrowth through the uncovered stent mesh or bleeding from the anchoring fins [9, 15]. On the technical aspects of biliary stenting, previous studies have evaluated the role of anchoring double-pigtail plastic stents (DPPS) into FCSEMS to prevent stent migration [16–18]. This review aims to explore the current evidence surrounding stent migration in dMBO and the efficacy of anchoring techniques by combining our own experience.

## Biliary stents for dMBO

PS are widely used for MBO due to their low cost and ease of insertion and removal. However, their shorter patency compared with SEMS makes them less ideal for long-term palliation in dMBO, leading to higher reintervention rates. Currently, PS are commonly used in patients with a life expectancy of <3 months or those scheduled for surgery [19, 20].

SEMS have become the standard of choice for dMBO management due to their superior patency compared with PS [21]. SEMS are broadly available in both uncovered and fully covered versions. In the early 2000s, studies showing longer patency in FCSEMS compared with UCSEMS led to widespread acceptance of the use of covered stents for dMBO [21, 22]. However, a published meta-analysis found that the duration of patency and survival did not differ between the two types of SEMS [6]. The risk of stent dislocation and sludge and biofilm formation are still debated issues in the choice of stent type.

## Stent migration of FCSEMS

The stent migration rates for FCSEMS range from 5% to 40% and dislocation can result in recurrent biliary obstruction, cholangitis, and the need for additional interventions, all of which negatively impact patient quality of life and delay cancer treatments [23–25]. Covered SEMS are coated with polyurethane, silicone, polytetrafluoroethylene, or Gore-Tex on the mesh to prevent the penetration of tumor tissue and mucosal hyperplasia, which actually reduces friction with the bile duct wall, increasing the chance of dislocation. Additionally, bile duct anatomy and chemotherapy-induced tumor shrinkage can influence migration rates. Tumor regression can lead to a decrease in the compressive effect of the stricture, further diminishing the hold of the stent [26].

The radial forces (RFs) and axial forces (AFs) of SEMS play crucial roles in both maintaining patency and influencing stent migration. Radial force is the outward pressure that the stent exerts against the bile duct walls, which helps to maintain patency and reduce the risk of occlusion. Although too much radial force can irritate the ductal wall, potentially leading to complications such as bile duct damage, low radial force causes insufficient stent expansion, which may cause cholangitis and early stent migration [26, 27]. Conversely, axial force—reflecting the flexibility of the stent along its length—allows the stent to conform to the natural curvature of the bile duct, but, if the axial force is high, then it can lead to poor conformability of the SEMS, resulting in bile duct kinking, acute cholecystitis, and pancreatitis due to bile duct wall, cystic duct, or pancreatic orifice compression [27, 28]. Commercially available biliary SEMS have mechanical properties according to the stent structure: braided-hook-and-cross-type (both low AF and RF), braided-cross-type (high AF and low RF), and laser-cut-type (intermediate AF and high RF) [28].

## Preventive strategies for stent migration

**Figure 1.**
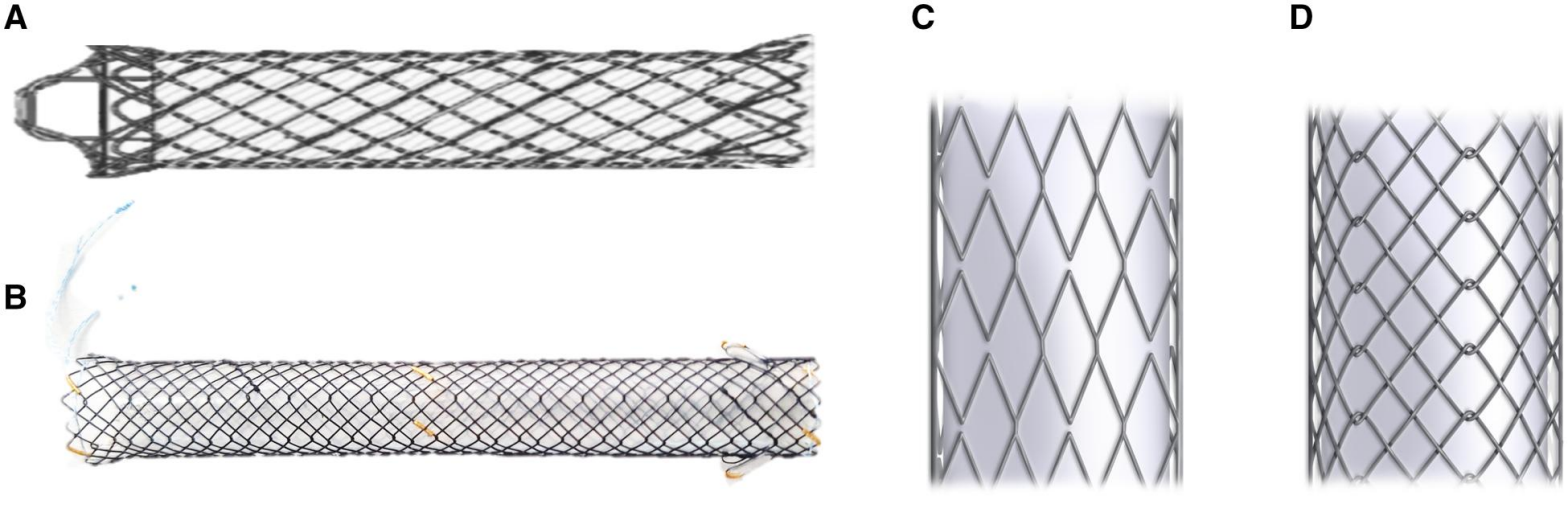
Types of biliary fully covered self-expandable metallic stents (FCSEMS). (A) FCSEMS with flared ends (WallFlex). (B) FCSEMS with anchoring flaps at the proximal end (HANARO). (C) Hook-and-cross type (braided structure). (D) Zigzag type (laser-cut structure). Courtesy of M.I. Tech Co. and Boston Scientific Co.

Several studies have demonstrated the anti-migration properties of different stent designs (Table 1). The anti-migration features of SEMS can be divided into two principal strategies: flared end and anchoring flap (Figure 1A and B). Isayama *et al.* developed laser-cut nitinol SEMS with flared ends and bank structures, and reported a 12.9% migration rate in patients with initial drainage and 70% (7 of 10 patients) prevention of migration in reintervention cases [10]. Petersen *et al.* also reported only one stent migration of 58 patients with braided SEMS with flared ends (WallFlex biliary RX stent, Boston Scientific, Natick, MA) [29]. In the comparison study with UCSEMS, a covered WallFlex stent showed longer patency (mean 219.3 vs 166.9 days; *P *=* *0.047) and stent migration was not observed [11]. Recently, Sakai *et al.* reported stent migration of 35.6%, which was a relatively high frequency of stent migration, for a single woven cross-structured nitinol wire-covered SEMS with flanged ends (Cook Evolution Biliary Stent; Cook Ireland Ltd) [14]. They explained that a high axial force may have contributed to the high frequency of stent migration.

**Table 1.** Migration rate of covered SEMS in previous studies

Study	Study design	Material	Construction	Anti-migration design	*N*	Migration rate	Stent patency (days), median
Isayama *et al.* (2013) [10]	Prospective	Modified covered ZEOSTENT	Laser-cut	Flared end with raised bank	31 (initial) 10 (reintervention)	4 (12.9%) 3 (30%)	107 176
Petersen *et al.* (2013) [29]	Prospective	WallFlex Biliary RX stent	Braided	Flared end	55	1 (1.8%)	210
Kitano *et al.* (2013) [11]	Prospective	WallFlex Biliary RX stent	Braided	Flared end	60	0 (0%)	219.3^a^
Sakai *et al.* (2024) [14]	Prospective	Cook Evolution Biliary stent	Braided	Flared end	73	26 (35.6%)	216
Kitagawa *et al.* (2022) [31]	Retrospective	ZEOSTENT covered stent WallFlex Biliary RX Partially covered stent	Laser-cut braided	Flare end	24 23	9 (37.5%) 3 (13.0%)	217 N/A^b^
Hasegawa *et al.* (2024) [32]	Prospective	ZEOSTENT covered stent WallFlex Biliary RX stent	Laser-cut braided	Flare and bank	29 31	6 (22.2%) 2 (6.8%)	220 418
Park *et al.* (2011) [9]	Prospective	HANARO flap stent BONASTENT biliary stent	Braided	Anchoring lap Flared end	22 21	0 (0%) 7 (33%)	N/A^c^
JiméNez-PéRez *et al.* (2014) [30]	Prospective	HANARO flap stent	Braided	Anchoring lap	64	1 (1.6%)	N/A^d^

a Mean value.

b Most patients died by the time the stent dysfunction occurred.

c Scheduled stent removal in benign biliary stricture.

d Stent patency not reported.

Anchoring flaps are alternative methods to prevent stent migration. Park *et al.* compared anti-migration effects of FCSEMS with an anchoring flap (M.I. Tech, Seoul, South Korea) to a flared end of the stent (Standard Sci Tech, Seoul, South Korea), and showed the superiority of the anti-migration effect (0% vs 33%) at 6 months after stent placement [9]. A study using an FCSEMS with four flaps at the proximal end (HANARO BCT stent; M.I. Tech, Seoul, Korea) reported one case of stent migration in 64 patients with dMBO [30]. Brinkmann *et al.* quantified the migration resistance of the three FCSEMS with flared ends and one FCSEMS with an anchoring flap [25]. Biomechanical measurements of the pull-out force revealed a 4-fold higher migration resistance of the anchoring flap and a lower rate of stent migration was observed compared with that of the flared end (5% vs 34%, *P *<* *0.001) [25].

As mentioned above, biliary SEMS can be divided into two types based on the fabrication methods (later-cut or braided) and have different mechanical properties of RFs and AFs (Figure 1C and D). Kitagawa *et al.* reported the efficacy of these two types of covered SEMS with anti-migration systems (ZEOSTENT covered; Zeon Medical, Tokyo, Japan for laser-cut-type and WallFlex Biliary RX Partially Covered Stent System; Boston Scientific, Marlborough, MA, USA for braided-type) for dMBO [31]. Braided-type covered SEMS showed significantly longer stent patency (*P *<* *0.001) and the major cause of stent dysfunction was stent migration, which was observed to be less in the braided-type (13% vs 37.5%) compared with the laser-type stent. A randomized–controlled trial comparing laser-cut-type ZEOSTENT or braided-type WallFlex Biliary RX FCSEMS demonstrated longer patency (220 vs 418 days, *P *=* *0.012) and lower stent migration (22.2% vs 6.8%) in braided-type FCSEMS [32]. One possible reason for the higher frequency of stent migration than expected is that the laser-cut ZEOSTENT loses anchoring force as it fully dilates due to relatively weak axial radial force than those of WallFlex PCSEMS and FCSEMS. Another drawback of the laser-type stent is difficulty in removing it at the time of reintervention [32].

Finally, the partially covered design of metal stents offers an alternative to FCSEMS by incorporating uncovered segments at their ends, which help to anchor the stent to the bile duct wall, thereby reducing migration risk [33, 34]. The recently published systematic review and meta-analysis by Vanella *et al.* included 62 studies (3327 with FCSEMS and 2322 with PCSEMS) and demonstrated a lower migration rate (4.3%) of PCSEMS compared with FCSEMS (9.8%) [35]. Although not statistically significant, the rate of tumor ingrowth was slightly higher (2.9% vs 0.5%) and the time to recurrent biliary obstruction was longer (369 vs 238 days) [35].

## Anchoring PS placement to FCSEMS

There are >10 different commercially available FCSEMS, varying in their anti-migration effectiveness, but institutions will not have access to all of them and endoscopists will choose stents based on factors other than anti-migration properties [21, 28]. Regardless of the type of stent, technically anchoring DPPS can prevent stent migration. Several studies have evaluated the role of internal (coaxial) or external (side-by-side) anchoring of DPPS to FCSEMS in preventing stent migration (Table 2). Park *et al.* anchored 5-Fr DPPS (Zimmon Biliary Stent; Cook Medical, Winston-Salem, NC) inside FCSEMS with a non-flared proximal end (S&G Biotech, Seongnam, Korea) for benign biliary strictures and compared it to the non-anchoring group [16]. Technical success of 100% was achieved in both groups. The stent migration rate was significantly lower (6.3% vs 41.2%, *P *=* *0.024) and the stent patency was significantly longer (154 vs 114 days, *P *=* *0.01) in the anchoring group than in the non-anchoring group. Emhmed *et al.* conducted a retrospective analysis to evaluate the anti-migration efficacy of internal anchored 7-Fr and 10-Fr DPPS in FCSEMS (WallFlex or VIABIL^®^) for malignant stricture (46.8%) or otherwise [18]. There was no significant difference in the stent migration rate in their study. However, the study had very heterogeneous indications, including not only malignant or benign strictures, but also post-sphincterotomy bleeding, cholangitis drainage, and bile leakage. Furthermore, no subgroup analyses were performed to determine the efficacy of anchoring DPPS in patients with MBO. Katsinelos *et al.* investigated the efficacy of 10-Fr DPPS to prevent stent migration in 10 patients with dMBO and 1 patient with a benign stricture [17]. During the median follow-up of 8 months (range 4–14), no migration of the FCSEMS was observed. The main drawbacks of their study are the small sample size and the non-randomized, single-arm study design.

**Table 2.** Studies with anchoring plastic stent in biliary stricture

Study	Design	*N*	Indications	Anchor	Migration rate	Stent patency (days)
Park *et al.* (2011) [9]	Prospective	37	Benign stricture	5Fr DPPS (internal anchoring)	Anchoring: 6.3% (1/16) Non-anchoring: 41.2% (7/17) *P *=* *0.024	154 114 *P *=* *0.010
Katsinelos *et al.* (2017) [17]	Prospective	11	Malignant stricture (10) Benign stricture (1)	10Fr DPPS (internal anchoring)	0%	N/A
Emhmed *et al.* (2019) [18]	Retrospective	203	Malignant stricture (95) Benign stricture (82) Others^a^ (26)	7Fr or 10 Fr DPPS (internal anchoring)	Anchoring: 6% (4/65) Non-anchoring: 10% (14/138) *P *=* *0.35	N/A
Paik *et al.* (2021) [15]	Prospective	68	Malignant stricture	7Fr DPPS 9 (internal anchoring)	Anchoring: 15% (5/33) Non-anchoring: 40% (14/35) *P *=* *0.35	237 173 *P *=* *0.048
Chun *et al.* (2023) [36]	Retrospective	185	Malignant stricture	7Fr DPPS (external anchoring)	Anchoring: 10.8% (13/120) Non-anchoring: 27.7% (18/65) *P *=* *0.01	342 240 *P *=* *0.04

a Others include Cholangitis drainage, bile leak, and post-sphincterotomy bleeding.

N/A = not applicable.

We assessed the value of placing internally anchored DPPS inside FCSEMS to help to reduce migration rates [15]. Seventy patients with unresectable dMBO were randomized to receive either 7-Fr DPPS with FCSEMS or FCSEMS alone. The technical success rate was 100% in both groups and the clinical success rates were similar (94% with anchoring of the PS and 83% without). The primary outcome of the study, namely the stent migration rate at 6 months, was significantly lower (15% vs 40%, *P *=* *0.02) and the stent patency was significantly longer (237 vs 173 days, *P *=* *0.048) in the anchoring group. The migration rate of FCSEMS was higher than that in previous reports. This may be due to the lower radial force of the FCSEMS (ARISTENT; Daewoong, Seoul, Korea) used in the study compared with other FCSEMS, the stent design without an anchoring flap, and tumor shrinkage after chemotherapy. In addition, the increased capture of stent migrations, even in asymptomatic patients, also affected the higher migration rate.

**Figure 2.**
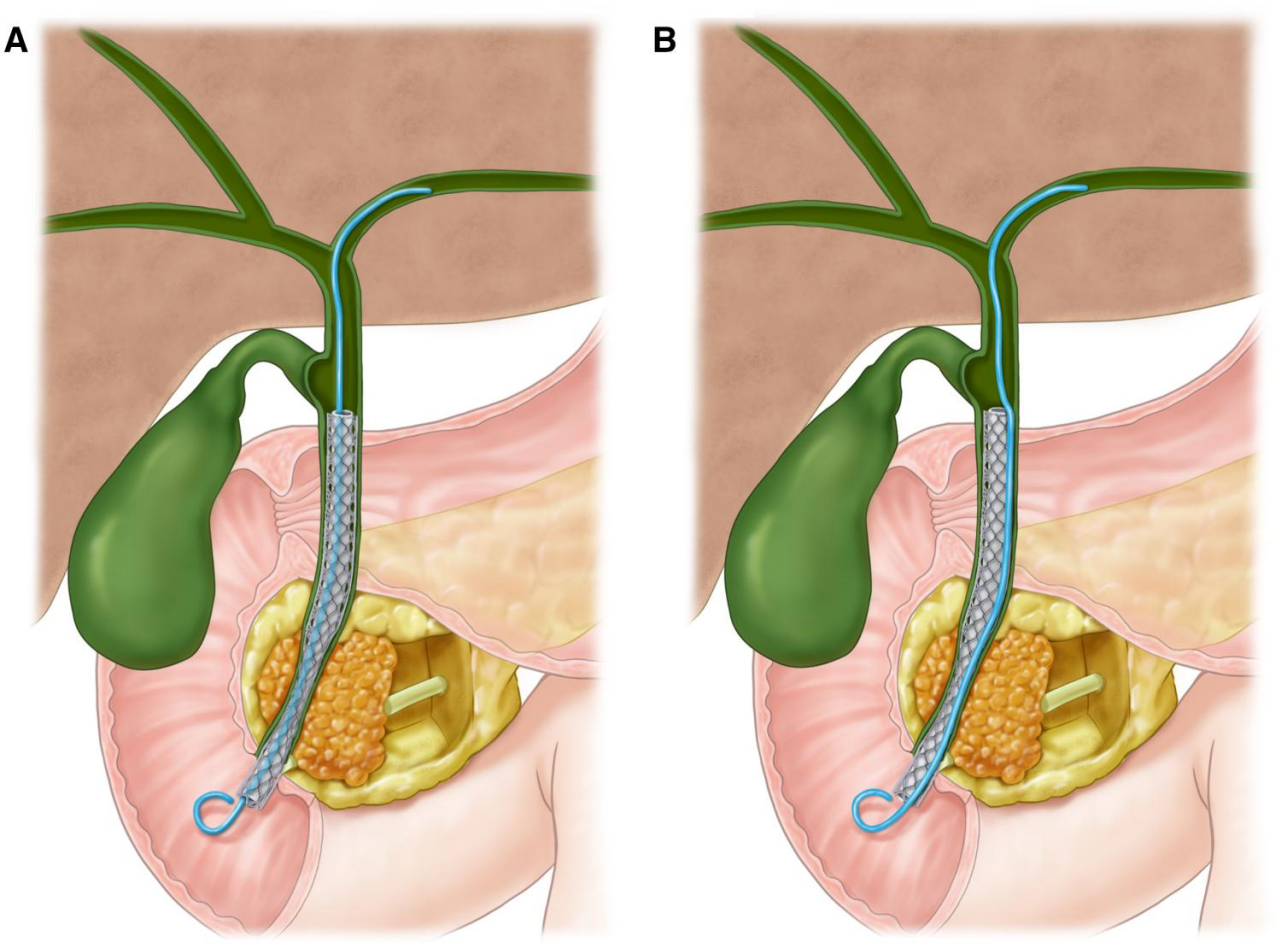
Anchoring DPPS technique. (A) Internal anchoring of DPPS inside fully covered self-expandable metallic stent. (B) External anchoring DPPS side-by-side placement with the fully covered self-expandable metallic stent.

The technique of anchoring PS can be easily implemented and effective in preventing stent migration. However, PS placed coaxially inside FCSEMS may be dislocated simultaneously with the FCSEMS migration, resulting in stent dysfunction. Therefore, we next evaluated the anti-migration efficacy of externally anchoring DPPS in patients with dMBO. Chun *et al.* retrospectively collected patients who had undergone endoscopic retrograde biliary drainage for dMBO [36]. Of the 185 patients, 65 had FCSEMS alone (Bonastent^®^ Biliary stent or ARISTENT) and 120 had FCSEMS with external 7-Fr DPPS anchoring. For external anchoring, two guide wires were positioned and DPPS were placed first, followed by side-by-side deployment of the FCSEMS (Figure 2B). During the follow-up, a significantly lower migration rate of the FCSEMS (11% vs 28%, *P *=* *0.01) was observed in the external anchoring group. The stent patency was also significantly longer (342 vs 240 days; *P *=* *0.04) in the FCSEMS with externally anchored DPPS than in the FCSEMS alone. Notably, in the external anchoring group, for 10 of 13 patients who had experienced migration, the DPPS remained in place, resulting in fewer stent revisions (8% vs 17%). Recently, Hawa *et al.* reported a systematic review and meta-analysis on the role of anchoring PS in biliary strictures. After reviewing 431 studies, they identified four eligible studies (three from South Korea and one from the USA), including two randomized–controlled trials and two retrospective studies. Their findings demonstrated that anchoring reduced the risk of FCSEMS migration by 67% compared with those without DPPS anchoring (odds ratio [OR] 0.33; 95% confidence interval [CI] 0.19–0.57; I-squared statistic [*I*^2^] = 0%) [37]. These results suggest that external anchoring of DPPS to FCSEMS is a simple and effective technique to prevent migration, with the advantage of maintaining patency with residual DPPS after FCSEMS migration.

## Other anti-migration strategies

As patient survival improves due to advancements in chemotherapy, the demand for stents with both sustained stent patency and adverse events, such as stent occlusion and migration, is increasing. Emerging technologies in biodegradable stents and surface modified stents may offer promising alternatives [38, 39]. Recently, magnesium and its alloys showed good biocompatibility as well as antitumor effects on cancer cells [40]. Additionally, Kobayashi *et al.* introduced a biliary stent with an anti-migration functional coating and multiple small holes [41]. These features lower the membrane tension, allowing the stent to anchor more securely to the surrounding tissue and thereby decrease the stent migration. Multi-hole SEMS showed the longest stent patency with less stent migration compared with FCSEMS (2.8% vs 15.8%) and less tumor ingrowth compared with UCSEMS (13.2% vs 42.4%) [42]. Takeda *et al.* also reported the anti-migration efficacy of multi-hole SEMS (HANAROSTENT Biliary Multi-hole NEO; M.I.Tech) compared with conventional FCSEMS (HANAROSTENT Biliary Full Cover NEO; M.I.Tech) [43]. Although stent migration was less frequently observed in the multi-hole SEMS group (0% vs 17.6%, *P *=* *0.03), the overall recurrent biliary obstruction rates were similar between the groups. Other various attempts to chemically and mechanically modify stents have been effective only in preclinical models and have limited clinical application [44, 45].

From the technical aspect, Wang *et al.* recently demonstrated the anti-migration effects of metal clip anchoring in a multicenter randomized–controlled study. By anchoring the distal retrieval loop or terminal end of the FCSEMS with a metal clip adjacent to the duodenal mucosa, stent migration was reduced (16.7% vs 30%, *P *=* *0.030) at 6 months [46]. However, this method requires additional equipment that is not commonly used during ERCP and may cause difficulties in stent removal.

In this sense, the PS anchoring method, which can effectively reduce migration through technical modification, has the advantage of being immediately clinically applicable. Based on our experience and research findings, we assume that external anchoring with DPPS not only reduces stent migration due to friction effects, but also makes spaces for cystic duct drainage. Compression of the cystic duct opening is a possible mechanism of cholecystitis in FCSEMS as opposed to UCSEMS [1]. In addition, stent migration is a potential weakness compared with UCSEMS. To evaluate the efficacy of the external anchoring of DPPS to FCSEMS compared with UCSEMS alone, we have conducted a prospective randomized comparative study and are awaiting the results (NCT05220475).

Endoscopic ultrasound-guided biliary drainage (EUS-BD) is an emerging technique that employs a transmural approach by using SEMS or lumen-apposing metal stents, unlike ERCP, which relies on transpapillary drainage. EUS-BD may be a safe and effective alternative if ERCP fails and as a first-line approach in dMBO [47, 48]. Stent migration is also a potential adverse event of EUS-BD and anchoring of the DPPS may be theoretically beneficial in preventing migration in EUS-BD [49].

## Conclusions

In managing dMBO, FCSEMS offer advantages in preventing tumor ingrowth and are more easily removable. However, they carry a higher risk of migration compared with UCSEMS. This review highlights various anti-migration strategies aimed at enhancing stent stability, including flared ends, anchoring flaps, and the use of DPPS as internal or external anchors. Studies indicate that both flared and anchoring flaps can significantly reduce migration, providing mechanical resistance within the bile duct. Attempts to modify the composition and structure of biliary stents and coating materials continue to be made. In addition, anchoring with internal or external PS has been shown in our studies to reduce migration rates in patients with dMBO. Ongoing research and clinical trials will be essential to refine these technologies and identify the most effective stent designs or techniques, ultimately improving outcomes for patients with dMBO.

## Authors’ contributions

S.H.L. contributed to the design of the review and the revision of the manuscript; J.W.C. collected the data and drafted the manuscript; W.H.P. and S.M.W. collected the data and revised the manuscript; I.R.C., J.H.C., W.J.L., J.K.R., and Y.T.K. contributed to the review and editing of the manuscript. All authors read and approved the final manuscript.
